# Engineering of chimeric peptides as antagonists for the G protein-coupled receptor, RXFP4

**DOI:** 10.1038/s41598-019-53707-z

**Published:** 2019-11-28

**Authors:** Praveen Praveen, Ross A. D. Bathgate, Mohammed Akhter Hossain

**Affiliations:** 1 Florey Institute for Neuroscience & Mental Health, University of Melbourne, Melbourne, VIC Australia; 2 Department of Biochemistry and Molecular Biology, University of Melbourne, Melbourne, VIC Australia; 3 School of Chemistry and Bio21, University of Melbourne, Melbourne, VIC Australia

**Keywords:** Chemical biology, Peptides

## Abstract

Insulin-like peptide 5 (INSL5) is a very important pharma target for treating human conditions such as anorexia and diabetes. However, INSL5 with two chains and three disulfide bridges is an extremely difficult peptide to assemble by chemical or recombinant means. In a recent study, we were able to engineer a simplified INSL5 analogue **13** which is a relaxin family peptide receptor 4 (RXFP4)-specific agonist. To date, however, no RXFP4-specific antagonist (peptide or small molecule) has been reported in the literature. The focus of this study was to utilize the non-specific RXFP3/RXFP4 antagonist ΔR3/I5 as a template to rationally design an RXFP4 specific antagonist. Unexpectedly, we demonstrated that ΔR3/I5 exhibited partial agonism at RXFP4 when expressed in CHO cells which is associated with only partial antagonism of INSL5 analogue activation. In an attempt to improve RXFP4 specificity and antagonist activity we designed and chemically synthesized a series of analogues of ΔR3/I5. While all the chimeric analogues still demonstrated partial agonism at RXFP4, one peptide (Analogue 17) exhibited significantly improved RXFP4 specificity. Importantly, analogue 17 has a simplified structure which is more amenable to chemical synthesis. Therefore, analogue 17 is an ideal template for further development into a specific high affinity RXFP4 antagonist which will be an important tool to probe the physiological role of RXFP4/INSL5 axis.

## Introduction

The gut endocrine system is a rich source of peptides with therapeutic potential^1–3^. The demonstrated involvement of altered gut hormone profiles in both the success of bariatric surgery and the treatment of metabolic disorders implies that greater understanding and informed manipulation of gut hormones may lead to meaningful therapies for conditions such as anorexia, obesity, and diabetes^4,5^.

**Figure 1:**
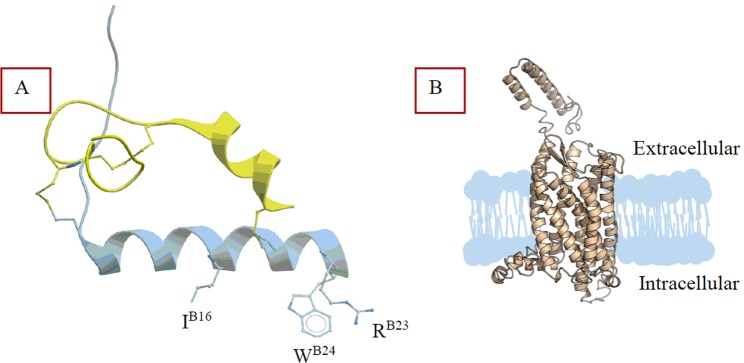
(**A**) NMR structure of human INSL5 (PDB: 2KBC) with primary binding residues in the B-chain (I^B16^, R^B23^, W^B24^) highlighted. The image was generated by Molsoft LLC software (ICM-browser Pro(x64); ActiveICM (version 1.2–4); www.molsoft.com). (**B**) Hypothetical structure of the RXFP4 receptor generated with Pymol.

Insulin-like peptide 5 (INSL5, Fig. 1A, Table 1) is a novel gut hormone produced by colonic L-cells^6^. Its target G protein-coupled receptor, Relaxin Family Peptide Receptor (RXFP4, Fig. 1B) is expressed in the enteric nervous system^6^. The first biological data in RXFP4 knockout mice suggested a potential role of INSL5 in appetite regulation^6^. However, the primary source of INSL5 comes from a gut region (colonic enteroendocrine L cells^6^) that has only a minor influence on feeding. Therefore, there is a debate on the physiological role of INSL5 and RXFP4. The recent data in the literature also suggest that INSL5/RXFP4 system may be a potential therapeutic target for Type 2 diabetes^7^.

**Table 1:**
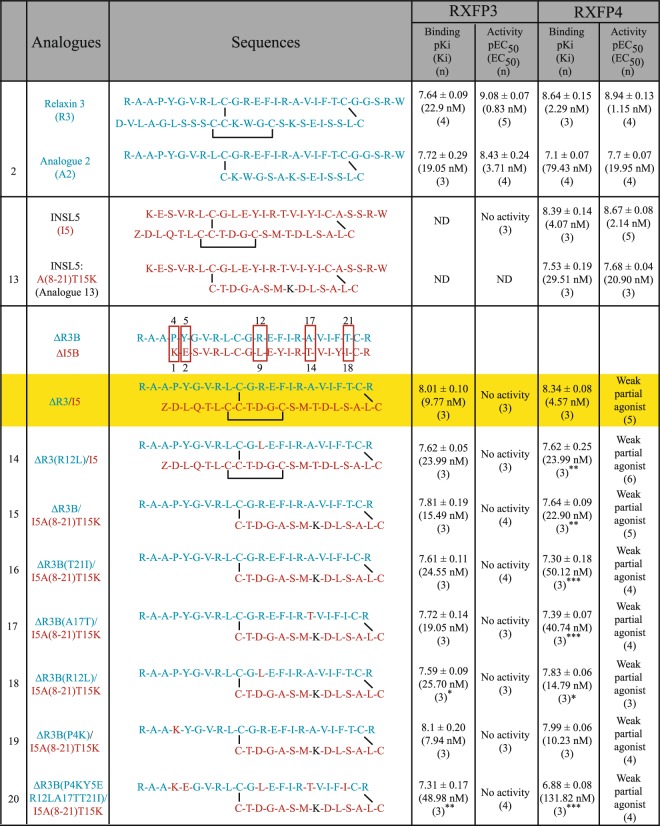
Primary structure of INSL5/H3 relaxin, their minimized analogues and chimeric peptides and their receptor binding affinity (pKi/Ki) and cAMP activation (pEC_50_/EC_50_) on the RXFP4/RXFP3 receptors.

The different amino acids in the B-chain of INSL5 and H3 relaxin with chemically distinct side chains are boxed. Z is pyroglutamate which is cyclic form of glutamic acid or glutamine that is produced by the loss of a water molecule. ***p < 0.001, **p < 0.01, *p < 0.05 vs ΔR3/I5.

Studies on the biology of INSL5 have been limited by the lack of sufficient quantities of the native peptide and the absence of small molecule RXFP4-specific agonists. Synthesis of both human INSL5 and mouse INSL5 has proven to be extraordinarily challenging by either chemical^8,9^ or recombinant means^10^. The A-chain is insoluble, and B-chain is aggregating in nature, and therefore, both synthesis and purification were found to be very difficult, resulting in very poor yield^8,9^. We recently undertook structure-activity relationship (SAR) studies on INSL5 and utilized the knowledge to engineer a simplified, potent RXFP4 agonist peptide, analogue **13** (Table 1)^11^. The peptide is an INSL5 analogue that has a simplified A-chain connected to the B-chain by two disulfide bonds and is readily synthesized in 17.5 fold higher yield than INSL5^11^. A small-molecule agonist was recently reported^12^ which has nanomolar potency both at RXFP4, and RXFP3, a receptor for human relaxin-3 (H3 relaxin)^12^. However, to date, no RXFP4-specific antagonist (peptide or small molecule) has been reported in the literature and such a compound would be enormously valuable to understand the physiology of INSL5 and validate RXFP4 as potential therapeutic target for the treatment of human conditions such as obesity and diabetes.

Some peptide ligands of GPCRs utilize amino acid residues for high affinity binding which are distinct from residues involved in activation. One way of generating antagonist peptide ligands is to delete the amino acids responsible for receptor activation while not affecting the binding interaction. This method was successful for developing antagonist for related receptors, RXFP2^13,14^ (endogenous receptor for insulin-like peptide 3, INSL3) and RXFP3^15,16^. This was possible as the amino acids involved in activation did not greatly contribute to binding affinity, and thus deletion of these residues resulted in receptor antagonists. However, extensive SAR studies on INSL5 to date have been unable to separate distinct binding and activation domains^17^. While R^B23^ and W^B24^ in INSL5 are responsible for RXFP4 activation in a similar manner to the same residues in H3 relaxin, in INSL5 these residues also contribute significantly to the RXFP4 binding interaction^17^. Therefore, here, we explored alternative rational design options to develop RXFP4-selective antagonists.

A chimeric peptide consisting of the A-chain of INSL5 and C-terminally truncated B-chain of H3 relaxin, ΔR3/I5 (Table 1), was reported in the literature a decade ago^16^. *In vitro* pharmacological studies showed that ΔR3/I5 is a high-affinity antagonist for both RXFP3 and RXFP4 and it has been used successfully in rodent brain studies to demonstrate specific roles of RXFP3 as RXFP4 is not expressed in the brain^16^. In this study we have utilized the ΔR3/I5 peptide as a template to rationally design an RXFP4 antagonist based on our understanding of RXFP3 and RXFP4 structure function relationships. Unexpectedly, ΔR3/I5 demonstrated weak partial agonism of cAMP activation in RXFP4 expressing cells which was associated with only partial antagonism of INSL5 analogue mediated cAMP activation. Rational design of synthetic ΔR3/I5 analogues demonstrated improvements in RXFP4 specificity however still demonstrated weak partial agonism associated with only partial RXFP4 antagonist activity.

## Results and Discussion

The human relaxin family is comprised of seven peptide hormones that include H1 relaxin, H2 relaxin, H3-relaxin, INSL3, INSL4, INSL5, and INSL6. H2 relaxin, H3-relaxin, INSL3 and INSL5 exert various biological functions through their GPCR receptors RXFP1-4, respectively^18^. While INSL5 is a selective ligand (agonist) for RXFP4, other family peptides are non-selective agonists for RXFP1-RXFP4. H3 relaxin, for example, is an endogenous ligand for RXFP3. However, it also strongly interacts with RXFP1 and RXFP4. To fully understand the role of relaxin family peptides, the development of selective agonists and antagonists is crucial. Recent reports have demonstrated that such receptor-selective agonists and antagonists can be achieved by making chimeric peptides^16,19,20^. ΔR3/I5 is an example of such a chimeric peptide. While H3 relaxin binds three receptors (RXFP1, RXFP3, and RXFP4), the chimeric ΔR3/I5 interacts with RXFP3 and RXFP4 only where it is a high affinity antagonist. This study reports further modification of ΔR3/I5 leading to improvements in RXFP4 selectivity.

### Design and synthesis of ΔR3/I5 and ΔR3/I5-based chimeric analogues

Instead of applying conventional alanine scanning to determine which residues in ΔR3/I5 are responsible for selectivity between RXFP3 and RXFP4, we have rationally designed and chemically assembled several chimeric analogues of INSL5 and H3 relaxin (Table 1) based on our knowledge of the INSL5/RXFP4 and H3 relaxin/RXFP3 interactions^18^. We synthesized seven ΔR3/I5-based analogues (**14–20**) and have utilized four previously reported peptides (H3 relaxin^21^, INSL5^8^, analogue **2**^22^, analogue **13**^11^ and ΔR3/I5^16^) as controls (Table 1). We have used H3 relaxin and analogue **2** as RXFP3 agonists, INSL5 and analogue **13** as RXFP4-selective agonists, and ΔR3/I5 as a non-selective antagonist for both RXFP3 and RXFP4 (Table 1). All the analogues were made with free C-termini (carboxylic acid) as we have previously shown that INSL5 and H3 relaxin analogues with amidated C-termini were less active compared with native INSL5 or H3 relaxin with free C-termini^9^. All linear single-chain peptides (A and B chains) were synthesized by Fmoc solid-phase peptide synthesis using automated peptide synthesizer (Liberty Blue) and then connected them by disulfide bonds in solution. The peptides were purified by reverse-phase high-performance liquid chromatography, and high final purity (93–99%) was confirmed by MALDI-TOF MS and RP-HPLC analysis (Supplementary Information). The concentration of peptide samples for pharmacological testing was confirmed by using Direct Detect assay-free sample cards and the Direct Detect spectrometer (Supplementary Information).

### ΔR3/I5 – a previous study

ΔR3/I5 was originally reported to strongly bind to RXFP3 and RXFP4 but showed no agonist activity (NA) at either of these receptors^16^ suggesting that ΔR3/I5 is an antagonist for both RXFP3 and RXFP4. The antagonism of RXFP3 and RXFP4 was compared, and the results showed that ΔR3/I5 dose-dependently shifted the agonism curves to the right confirming ΔR3/I5 to be an antagonist for both RXFP3 and RXFP4^16^. Here we have chemically synthesized and pharmacologically characterized ΔR3/I5 for binding and cAMP potency in CHO cells stably expressing RXFP3 (CHO-RXFP3) or RXFP4 (CHO-RXFP4).

### The pharmacological activity of ΔR3/I5 at RXFP3

**Figure 2:**
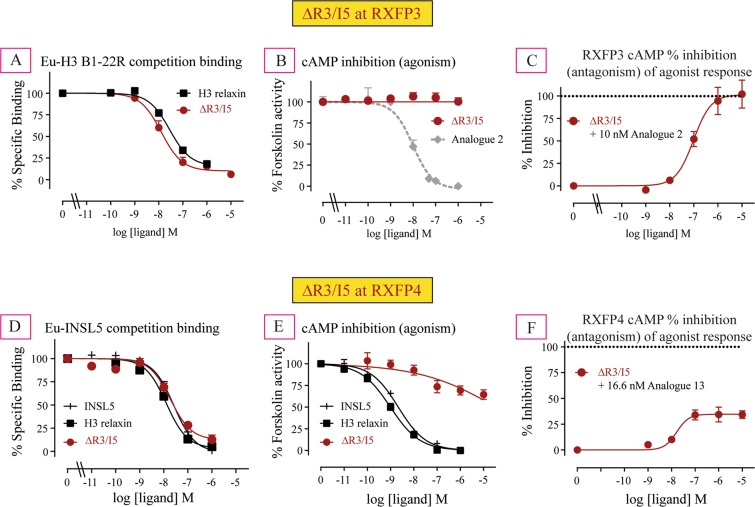
Binding, agonist and antagonist activities of ΔR3/I5 in RXFP3 cells. (**A**) Competition binding curve of ΔR3/I5 competing with 5 nM of Eu-B1–22R; (**B**) Agonist activity of ΔR3/I5 as measured by cAMP changes; (**C**) Ability of ΔR3/I5 to antagonize 10 nM analogue **2**-induced inhibition of cAMP activity. Binding, agonist and antagonist activities of ΔR3/I5 in RXFP4 cells. (**D**) Competition binding curve of ΔR3/I5 competing with 5 nM of Eu-INSL5; (**E**) Agonist activity of ΔR3/I5 as measured by cAMP changes; (**F**) Ability of ΔR3/I5 to antagonize 16.6 nM analogue **13**-induced inhibition of cAMP activity. The data are the result of n = 3–4 independent experiments and are expressed as mean ± SEM.

We have first tested our synthetic ΔR3/I5 in competition binding assays in CHO-RXFP3 cells. ΔR3/I5 exhibited high affinity binding at RXFP3 with an affinity higher than the native ligand H3 relaxin as previously reported (Ki of 9.77 nM compared to 22.9 nM; Table 1, Fig. 2A). The ΔR3/I5 was then tested for its ability to activate the RXFP3 receptor as measured via its ability to inhibit forskolin-stimulated cAMP production in CHO-K1-RXFP3 cells (Fig. 2B). Consistent with the previous report, ΔR3/I5 was unable to activate RXFP3 (Fig. 2B, Table 1) and displayed potent antagonistic activity (IC_50_ of 117.49 nM, Emax = 100%; Table 2, Fig. 2C) at RXFP3 evident from its ability to block 10 nM analogue **2** (RXFP3 agonist) inhibition of the forskolin-induced cAMP activity.

**Table 2:**
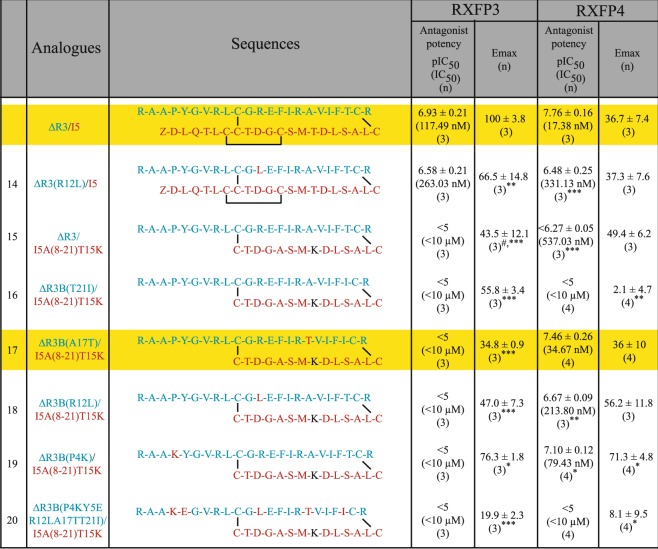
Primary structure of ΔR3/I5 and novel chimeric peptides and their antagonist potency (pIC_50_/IC_50_) and efficacy on the RXFP4/RXFP3 receptors.

***p < 0.001, **p < 0.01, *p < 0.05 vs ΔR3/I5.

### The pharmacological activity of Δ**R3/I5 at RXFP4**

We tested ΔR3/I5 in competition binding assays on human CHO-K1-RXFP4 cells. ΔR3/I5 showed high affinity binding to RXFP4 with an affinity similar to the native agonist INSL5 (Ki of 4.57 nM compared to 2.14 nM; Table 1, Fig. 2D). However, to our surprise, ΔR3/I5 acted as a weak partial agonist at RXFP4 (Table 1, Fig. 2E). This result seems to contradict the original published report where ΔR3/I5 was described as having no activity at human RXFP4 although dose response curves were not presented^16^. Notably, the original study utilized SK-N-MC/CRE-β-gal cells expressing human RXFP4 hence although the reporter gene system was similar the studies were done in a completely different cell system. Importantly, consistent with this partial agonist activity, antagonism assays utilizing synthetic ΔR3/I5 demonstrated that the peptide was only able to partially block INSL5 agonist mediated cAMP activation (Emax = 36.7% of response, IC_50_ of 17.38 nM, Table 2, Fig. 2F).

This is the first report of ΔR3/I5 showing partial agonism and antagonism of cAMP activation at human RXFP4. Notably, although ΔR3/I5 shows no agonist activity and is a full antagonist of H3 relaxin in CHO-RXFP3 cells utilizing the CRE-reporter gene assay^23^ and in SK-N-MC/CRE-β-gal cells expressing human RXFP3^16^ two other reports have shown that ΔR3/I5 is a partial agonist of RXFP3. Kocan *et al*. demonstrated that ΔR3/I5 was a weak partial agonist of p38MAPK, ERK1/2 and cAMP signalling but also was able to act as an antagonist of these pathways^24^. Another study demonstrated that ΔR3/I5 is a partial agonist in assays of [35 S]-GTPγS binding, cAMP activation and dynamic mass redistribution (DMR) in HEK-293s cells expressing rat and human RXFP3^25^.

In this study we have attempted to modify the ΔR3/I5 peptide to achieve RXFP4-specificity and improve the peptides antagonist properties at RXFP4.

### Novel ΔR3/I5-based analogues

Η3 relaxin interacts with both RXFP3 and RXFP4 by utilizing the same primary binding residues in the B-chain^16^: R^B8^, R^B12^, I^B15^, R^B16^, and F^B20^. INSL5, on the other hand, interacts with RXFP4 by utilizing primary binding residues in the B-chain: I^B16^, R^B23^ and W^B24^ in the B-chain (Fig. 1B)^17,26^. Given that H3 relaxin interacts with both RXFP3 and RXFP4 receptors and INSL5 interacts only with RXFP4 using B-chain specific residues, we speculated that we would be able to rationally design RXFP4-selective antagonist by modifying the B-chain of ΔR3/I5. Our strategy was to replace non-conservative residues of the B-chain of ΔR3/I5 with the corresponding residues of the B-chain of INSL5. We have compared the amino acid sequences of C-terminally deleted H3 B-chain (ΔH3B) in ΔR3/I5 with that of equivalent INSL5 B-chain (ΔINSL5B) (Table 1). There are 23 residues in the ΔH3B and 21 residues in the corresponding ΔINSL5B. However, of these, five residues involve differences with chemically distinct side chains (boxed in Table 1). We systematically replaced those five distinct residues in the B-chain of ΔR3/I5 with the corresponding residues of INSL5B.

### Binding of ΔR3/I5-based analogues at RXFP3 and RXFP4 cells

#### Three disulfide bond containing ΔR3/I5-based analogue **14**

**Figure 3:**
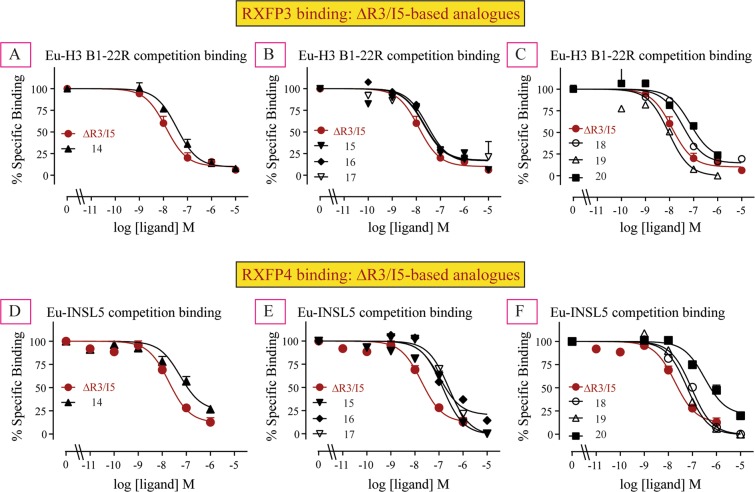
Competition binding curves for ΔR3/I5 and novel chimeric analogues **14–20**, (**A**–**C**) in comparison to Eu-B1-22R in RXFP3-expressing cells and (**D**–**F**) in comparison to Eu-INSL5 in RXFP4-expressing cells. The data are the result of n = 3–4 independent experiments and are expressed as mean ± SEM.

R^B12^ in the B-chain of ΔR3/I5 was previously reported to be one of the residues important for binding to both RXFP3 and RXFP4^16^. The corresponding residue L^B9^ in the B-chain of INSL5, on the other hand, was reported to be one of the most critical residues by which INSL5 exhibits selectivity for RXFP4 over RXFP3^27^. Therefore, in the first attempt, we replaced the R^B12^ residues in the B-chain of ΔR3/I5 with corresponding L^B9^ residue of INSL5 B-chain in order to achieve selectivity for RXFP4 over RXFP3 (Table 1). To our surprise, the resulting analogue **14** did not show significantly reduced affinity for RXFP3 (Ki of 23.99 nM; Table 1, Fig. 3A), but did show significantly less affinity for RXFP4 (Ki of 23.99 nM; Table 1, Fig. 3D) compared with ΔR3/I5. The fact that the agonist peptide R3/I5 with the same mutation (R^B12^/L^B9^) was shown to exhibit ~1600 less RXFP3 potency without losing much RXFP4 potency^27^ suggest that the binding mode of the agonist (R3/I5) peptide for RXFP3 and RXFP4 might be different to that of the antagonist peptide (ΔR3/I5).

#### Two disulfide bond containing ΔR3/I5-based analogue **15**

The current lead antagonist ΔR3/I5 or mutated analogue **14** has two chains (A and B chain) and three disulfide bonds with 43 residues (Table 1). Chemical synthesis of this peptide is tedious, time-consuming, and low yielding (3.3%) (Supplementary Information). We therefore decided to simplify the structure of ΔR3/I5 so that we can facilitate SAR studies. In this context, we replaced the A-chain (21 residues) of ΔR3/I5 with the modified A-chain (14 residues) of the simplified RXFP4 agonist, analogue **13**^11^ (Table 1). The resulting analogue **15** has only two-disulfide bridges and is high yielding (17.4%) (Supplementary Information). Excitingly, analogue **15** showed high affinity for both RXFP3 (Ki of 15.49 nM; Table 1, Fig. 3B) and RXFP4 (Ki of 22.90 nM; Table 1, Fig. 3E). Therefore, we have used this high yielding chimeric peptide template **15** for further SAR studies as outlined below.

#### Two disulfide bond containing ΔR3/I5-based analogues **16–20**

The analogues (**16–19**) derived from analogue **15** by single amino acid replacement showed strong binding affinity for both RXFP3 (Ki of 7.94 nM to 25.70 nM; Table 1, Fig. 3B,C) and RXFP4 (Ki 10.23 nM to 50.12 nM; Table 1, Fig. 3E,F). When compared with analogue **15**, it is clear that non-conservative residues between the ΔB-chain of INSL5 and H3 relaxin have minimal or no contribution to RXFP3/RXFP4 binding. However, when all five distinct residues in the B-chain of analogue **15** were replaced with corresponding residues of the INSL5 B-chain, the resulting analogue **20** showed significantly less affinity compared with ΔR3/I5 for both RXFP3 (Ki of 48.98 nM; Table 1, Fig. 3C) and RXFP4 (Ki of 131.82 nM; Table 1, Fig. 3F) that might be due to accumulated loss contributed by each mutation or due to destabilization of the overall structure.

### Agonism potency of ΔR3/I5-based analogues at RXFP3 and RXFP4 cells

**Figure 4:**
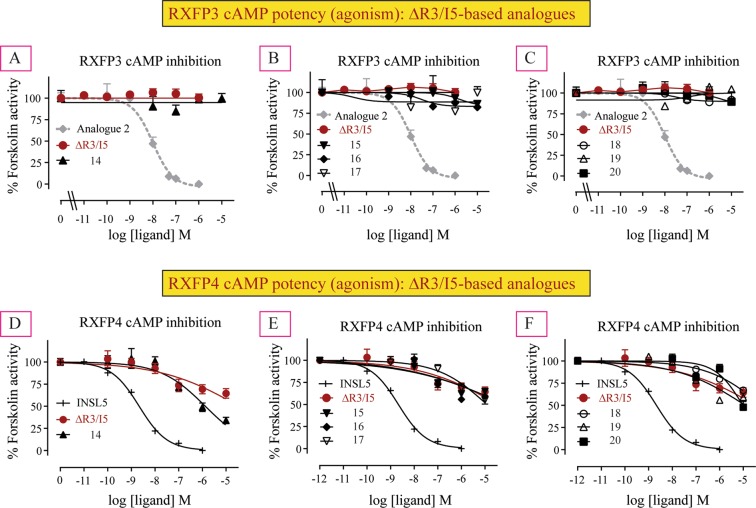
Agonist activities of novel chimeric analogues **14–20** compared with ΔR3/I5 in RXFP3 cells (**A**–**C**) and RXFP4 cells (**D**–**F**) as measured by changes in cAMP activity. The data are the result of n = 3–4 independent experiments and are expressed as mean ± SEM.

Like ΔR3/I5, all the analogues (**14–20**) demonstrated no significant activation of RXFP3 (Table 1, Fig. 4A–C) but acted as weak partial agonists at RXFP4 (Table 1, Fig. 4D–F) suggesting that they are likely to be antagonists for both RXFP3 and RXFP4.

### Antagonism potency of ΔR3/I5-based analogues at RXFP3 and RXFP4 cells

**Figure 5:**
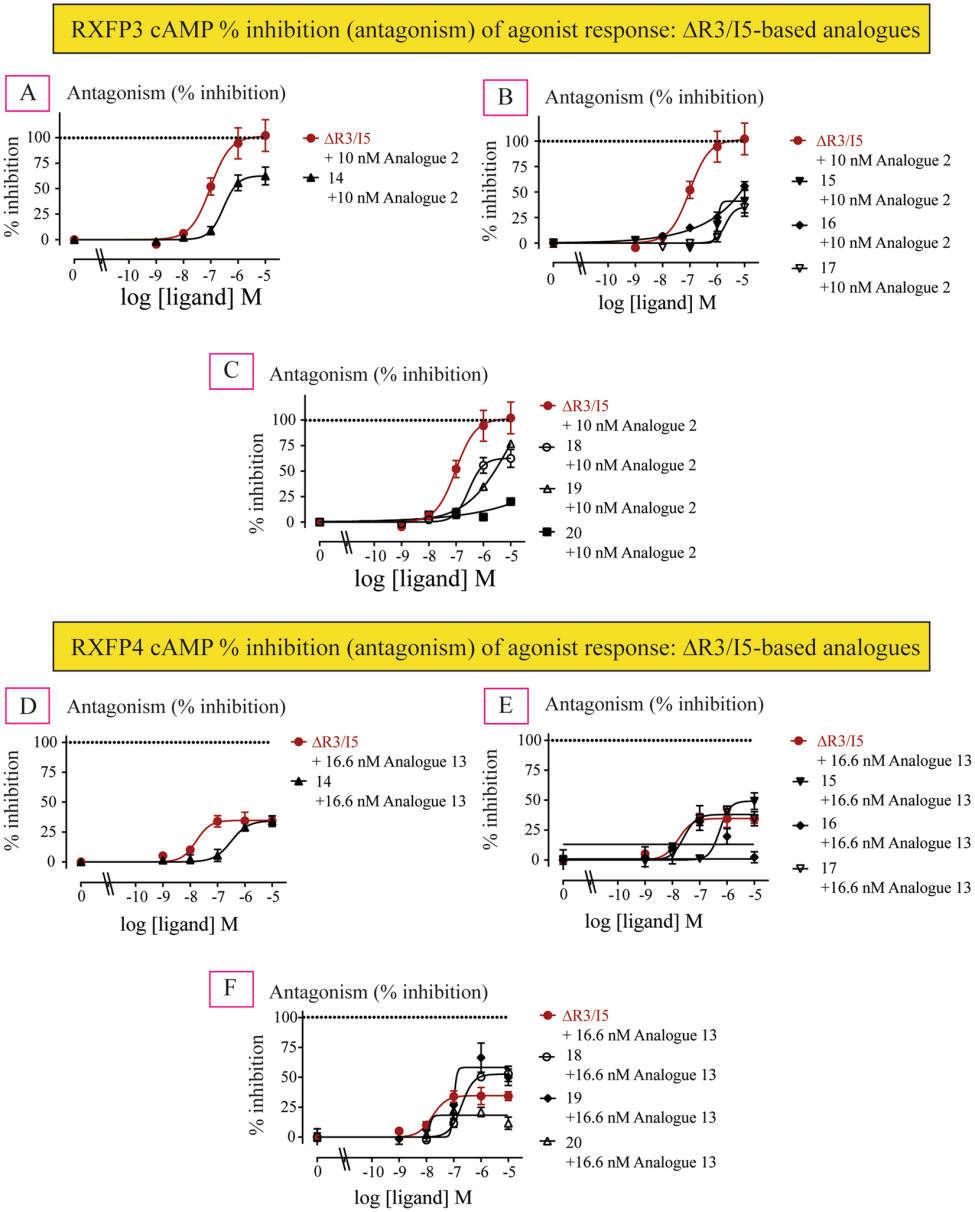
Antagonist activities of novel chimeric analogues **14–20** compared with ΔR3/I5 in RXFP3 and RXFP4 cells. (**A**–**C**) Ability of ΔR3/I5 and analogues **14–20** to antagonize 10 nM analogue **2**-induced inhibition of cAMP activity. **(D**–**F**) Ability of ΔR3/I5 and analogues **14–20** to antagonize 16.6 nM analogue **13**-induced inhibition of cAMP activity. The data are the result of n = 3–4 independent experiments and are expressed as mean ± SEM.

**Figure 6:**
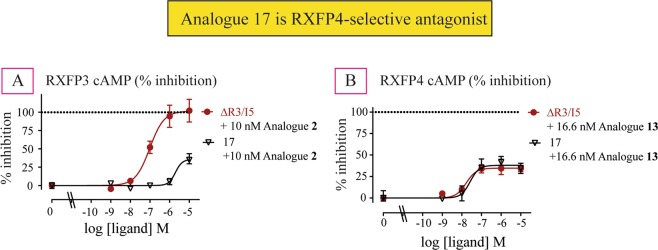
Antagonist activity of novel chimeric analogue **17** compared with ΔR3/I5 in RXFP3 and RXFP4 cells. (**A**) Ability of ΔR3/I5 and analogues **17** to antagonize 10 nM analogue **2**-induced inhibition of cAMP activity. (**B**) Ability of ΔR3/I5 and analogues **17** to antagonize 16.6 nM analogue **13**-induced inhibition of cAMP activity. The data are the result of n = 3–4 independent experiments and are expressed as mean ± SEM.

Interestingly, despite high binding affinity and no activity at RXFP3, all the analogues (**14–20**) showed significantly less antagonistic potency and efficacy (Fig. 5A–C, Table 2) compared with ΔR3/I5. This result was initially encouraging as it suggested some RXFP4-selectivity. However, when tested in RXFP4 cells, all analogues except two (**17** and **19**) showed less antagonistic potency compared with ΔR3/I5 (Fig. 5D–F, Table 2). Interestingly, analogues **17** and **19** showed high antagonistic potency (**17**: IC_50_ of 34.67 nM, **19**: IC_50_ of 79.43 nM; Table 2; Fig. 5E,F) with analogue **19** showing higher efficacy (Emax of 71.3%) than ΔR3/I5. Importantly, while analogue **17** has ΔR3/I5-like antagonist potency for RXFP4, it was found to be more selective for RXFP4 over RXFP3 (Fig. 6, Table 2) compared with both ΔR3/I5 and analogue **19**. However, all of the chimeric ΔR3/I5 analogues still demonstrated partial agonist activity like the parent ΔR3/I5 peptide and importantly were only able to partially antagonize RXFP4 agonist activation. Nonetheless, analogue **17** demonstrates improved selectivity for RXFP4 and has a simpler structure compared with ΔR3/I5 which enables its preparation in higher yield (yield of ΔR3/I5 = 3.3% and yield of analogue **17** = 17.4%; Supplementary Information)

### Structural study of ΔR3/I5 and analogue 17

**Figure 7:**
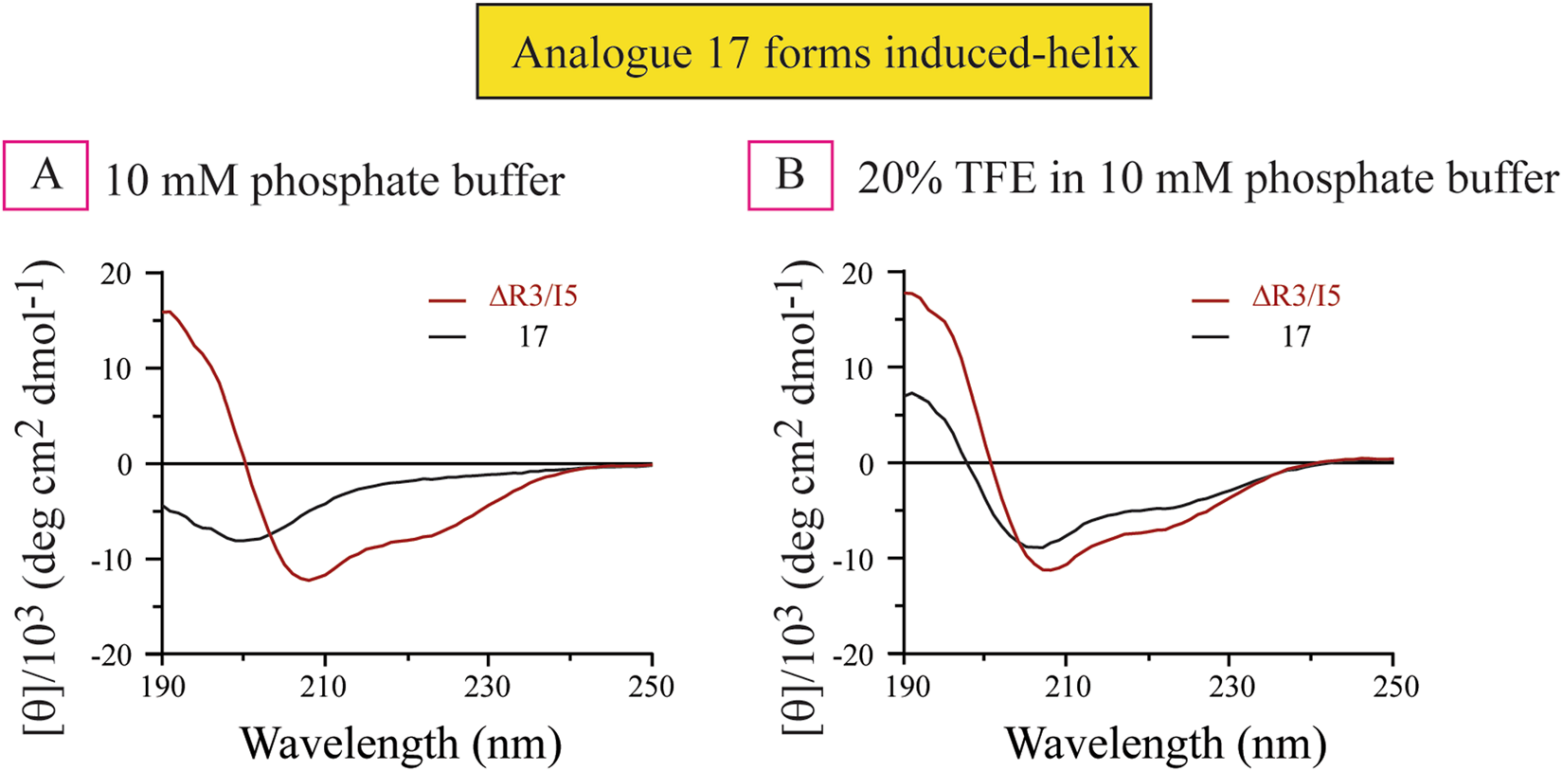
CD spectrum of analogue **17** compared with ΔR3/I5. CD was performed in 10 mM PBS buffer at pH 7.5 (**A**) and in 20% TFE (**B**).

While the structure of the ΔR3/I5 peptide has not been determined, the NMR solution structure of the agonist peptide R3/I5 has been solved^28^. The NMR solution structure of R3/I5 revealed that the INSL5 A-chain adopts a conformation similar to the H3 relaxin A-chain, and thus can structurally support a native-like conformation of the H3 relaxin B-chain through which it interacts with both RXFP3 and RXFP4. Truncations of the A-chain and/or removal of the intra-A-chain disulfide bond have been previously used to successfully simplify the structure while maintaining affinity and efficacy of several relaxin peptides^11,22,29,30^. However, extensive truncation or removal of the disulfide bond often leads to a compromised overall structure^29,30^. Such minimized peptides are generally challenging to study by NMR due to the increased structural flexibility resulting in spectral line broadening^31^ and tendencies of aggregation^32^. To investigate the conformation of analogue **17** in solution, and to compare it to that of ΔR3/I5, we therefore decided to carry out circular dichroism (CD) studies. Consistent with the solution NMR structure of R3/I5, our synthetic ΔR3/I5 demonstrated clear α−helical structure in aqueous buffer with characteristic double minima in the spectrum at 208 and 222 nm (Fig. 7A). In contrast, analogue **17** showed spectra representing principally random coil structure (Fig. 7A). As analogue **17** has strong RXFP4 binding affinity (IC_50_ of 34.67 nM), we postulate that analogue **17** adopted an appropriate conformation upon binding to the receptor. To confirm this hypothesis, we have studied CD spectroscopy of these peptides in 20% trifluoroethanol (TFE) in phosphate buffer. Trifluoroethanol (TFE) is known to stabilize the α−helical structure of peptide and proteins by improving hydrogen bonding. While 20% TFE did not significantly affect the secondary structure of ΔR3/I5 (Fig. 7B), it induced helical structure in analogue **17**, suggesting that analogue **17** has a tendency to form a helix. This finding is consistent with our previous studies where the truncated INSL5 analogues **6**^30^ and **13**^11^ exhibited compromised fold but showed very high RXFP4 affinity^30^. A similar trend was also observed for a H3 relaxin analogue where the A-chain was truncated up to 9 residues at the N-terminus, and the resulting analogue with compromised structure was found not to significantly affect binding or activation of RXFP3^29^.

## Conclusion

In conclusion, we have demonstrated for the first time that the RXFP3/RXFP4 antagonist ΔR3/I5 exhibits partial agonism at RXFP4 when expressed in CHO cells which is associated with only partial antagonism of INSL5 analogue activation. In an attempt to develop an RXFP4-specific antagonist we have rationally designed and chemically synthesized a series of simplified analogues of ΔR3/I5. All of the chimeric analogues still demonstrated partial agonism at RXFP4, however analogue **17** exhibited improved RXFP4 specificity. Moreover, analogue **17** has a simplified structure which enables its synthesis in higher yields than ΔR3/I5. Therefore, analogue **17** is a potential template for further SAR to improve RXFP4 antagonistic efficacy and facilitate pre-clinical studies to probe the physiological role of RXFP4/INSL5 axis.

## Methods

### Peptide synthesis

All the polypeptide chains were assembled on preloaded resins using Fmoc solid phase synthesis, and our optimized synthesis protocols^11^ on the microwave assisted liberty peptide synthesizer (CEM liberty, Mathew, USA). The side chain-protecting groups of trifunctional amino acids were TFA-labile, except for tert-butyl (tBu)-protected and acetamidomethyl (Acm)-protected cysteine Cys in the A-chain and Cys (Acm) in the B-chain. The peptides were synthesized on a either 0.1 mmol scale using instrument default protocols with either a 4-fold molar excess of Fmoc-protected amino acids (0.4 mmol for a 0.1 mmol scale; 1.0 mmol for a 0.25 mmol scale) that were activated by using 3.8-fold excess of HCTU in the presence of excess of diisopropylethelene amine (DIEA). N^α^-Fmoc protecting groups were removed by treating the resin-attached peptide with piperidine (20% v/v) in DMF. Using the microwave synthesizer, the coupling and deprotection were carried out at 75 °C using 25 W microwave power for 5 min and 60 W microwave power for 5 or 3 min respectively. For manual synthesis the coupling and deprotection were carried out for 60 min and 5 min 3 times respectively.

### Peptide purification and characterization

We have analyzed, purified and characterized the synthetic peptides by using reversed-phase high performance liquid chromatography (RP-HPLC) and matrix-assisted laser desorption ionization time-of-flight mass spectrometry (MALDI-TOF MS) (Supplementary Information).

### Peptide characterization by amino acid analysis

The peptide content was determined using Direct Detect assay-free sample cards and the Direct Detect spectrometer. Each card contains hydrophilic spots surrounded by a hydrophobic ring to retain the analyzed sample within the IR beam for convenient sample application and analysis. All measurements were performed using 2 μL of sample solution.

### Peptide analogues prepared and characterized

**Human INSL5, H3 relaxin (R3), ΔR3/I5** and analogue **14**: These peptides have two chains (A and B) with three disulfide bonds (Table 1). Both the individual A- and B-chains were obtained in high yields and purity. We have used orthogonal protecting groups for cysteine residues. Each of the three disulfide bond was formed as per our previously reported protocol^8,9^. The analogues were identified by MALDI-TOF MS. Previously reported control peptide ΔR3/I5 was made for this study and others controls **H3 relaxin** and **INSL5** were previously made and used for this study. Δ**R3/I5**: m/z 4847.747 [M + H]^+^, calcd. 4851.71; analogue **14** m/z 4807.084 [M + H]^+^, calcd. 4808.69 (Supplementary Information).

Minimized analogues **2, 13**, **15, 16, 17, 18, 19** and **20**: We have used orthogonal protecting groups for cysteine residues and made two disulfide bridges between the A-and B-chain using our recently developed protocol^11,30^ that resulted in **15**, **16**, **17, 18, 19** and **20**. The analogues were identified by MALDI-TOF MS; **2:** m/z 4545.262 [M + H]^+^, calcd. 4537; **13**: 4263.929 [M + H]^+^, calcd. 4257; **15**: m/z 4064.187 [M + H]^+^, calcd. 4064.8; **16**: m/z 4084.635 [M + H]^+^, calcld. 4081; **17**: m/z 4102.015 [M + H]^+^, calcld. 4099; **18**: m/z 4020.433 [M + H]^+^, calcld. 4021.77; **19**: m/z 4102.603 [M + H]^+^, calcld. 4100; **20**: m/z 4066.955 [M + H]^+^, calcld. 4065. Previously reported analogues **2** and **13** were chemically prepared for this study (Supplementary Information). The purity of each of the peptides (≥95%) was determined by using analytical RP-HPLC peak area integration (Supplementary Information).

### Functional assays

#### Binding assays

Chinese hamster ovary CHO-K1 cells (sourced from American Type Culture Collection (ATCC), Maryland, USA) stably transfected with RXFP3^33^ or RXFP4^9^ were plated out onto precoated poly-L-lysine 96-well view plates at a density of 50 000 cells per well. Medium was aspirated off and the cells were washed with phosphate-buffered saline (PBS) before competition binding assays were performed with 5 nM Eu-H3B1–22R^34^ (RXFP3 ligand) or 5 nM Eu-DTPA-mINSL5^9^ (RXFP4 ligand) as previously described. Competition binding curves were performed in triplicate and each experiment was performed independently at least three times. Fluorescent measurements were carried out on a BMG POLARstar plate reader (BMG Labtech, Melbourne, Australia. Pooled data are presented as mean ± S.E of specific binding and are fitted using one-site binding curve in GraphPad Prism version 8 (GraphPad Inc., San Diego, USA). Statistical analyses were conducted using one-way analysis of variance with uncorrected Fisher’s least significant difference (LSD) post-hoc analysis in GraphPad Prism version 8.

#### cAMP (agonism or antagonism) assays

The peptides were tested for their ability to inhibit forskolin induced cAMP activity in CHO-K1-RXFP3 or CHO-K1-RXFP4 cells transfected with a pCRE (cAMP Response Element) β-galactosidase reporter plasmid as previously described^9,34^. For agonist assays cells were stimulated with forskolin (5 µM for RXFP3, 1 µM for RXFP4) plus or minus increasing concentrations of each peptide for 6 hours. For antagonist assays cells were stimulated with forskolin as in agonist assays plus increasing concentrations of each peptide for 10 minutes followed by stimulation with agonist at an ~EC80 concentration for a further 6 hours (10 nM Analogue 2 for RXFP3 and 16.6 nM Analogue 13 for RXFP4). Each peptide was tested in triplicate and each experiment was performed independently at least three times. Agonist data was expressed as the % Forskolin activity whereby 100% was defined as Forskolin alone and 0% as maximum agonist stimulation (1 µM Analogue 2 for RXFP3 and 1 µM Analogue 13 for RXFP4). Antagonist data was expressed as % inhibition whereby 0% was defined as agonist alone and 100% Forskolin alone. Data were analysed and plotted using GraphPad PRISM 8 and are expressed as the mean ± SEM of the pooled data. Statistical analysis was conducted using one-way ANOVA with Uncorrected Fisher’s LSD post-hoc analysis in GraphPad Prism version 8.

### Circular dichroism (CD) spectroscopy

CD spectra data of all peptides were recorded using a JASCO J-800 spectrophotometer at 25 °C using 1 mm path length cell. The peptides were dissolved in 10 mM phosphate buffer at pH 7.5. The parameters used to obtain the spectra were wavelengths 190 to 260 nm with a data pitch of 0.1 nm, continuous scanning mode at a speed of 50 nm per minute and the number of accumulations taken per peptide was 3. The concentration of peptide used was 0.2 µg/µl.

## Supplementary information

Supplementary information
