# Supplementary material for: Engineering of chimeric peptides as antagonists for the G protein-coupled receptor, RXFP4
Source: Sci Rep. 2019 Nov 28;9:17828. doi: 10.1038/s41598-019-53707-z (PMC6882824; doi:10.1038/s41598-019-53707-z)
Supplement: Supplementary file 1 — Supplementary information [file 41598_2019_53707_MOESM1_ESM.pdf]

-

SUPPLEMENTARY INFORMATION

Engineering of chimeric peptides as antagonists for the G protein-coupled receptor, RXFP4

Praveen Praveen[†], Ross A. D. Bathgate[†Δ*], and Mohammed Akhter Hossain[†‡*]

[†]Florey Institute for Neuroscience & Mental Health, [Δ]Department of Biochemistry and Molecular Biology, [‡]School of Chemistry and Bio21, University of Melbourne, VIC, Australia

Address correspondence to:

A/Prof Mohammed Akhter Hossain

Email: akhter.hossain@unimelb.edu.au

Tel: +61 3 83440414

&

Professor Ross Bathgate

Email: bathgate@florey.edu.au

Tel: +61 3 90356735

Florey Institute of Neuroscience & Mental Health,

University of Melbourne,

Parkville

Victoria 3010

Australia

-

Table of Contents

1 Analytical RP-HPLC and MALDI-TOF-MS of all compounds S2-S7
2 Table S1: Summary of peptide characterization by HPLC and MALDI TOF MS S8
3 Peptide % Yield S8
4 References.. S8

1. Peptide characterization by RP-HPLC and MALDI

Analytical RP-HPLC analysis of the purified compounds was performed with Waters RP-HPLC systems with Empower software for data collection, monitoring and analysis. The RP-HPLC profiles were acquired using a Phenomenex Gemini C18 analytical column (4.6 x 250 mm, pore size 300 Å, particle size 5 µm), at a constant flow rate of 1.5 mL/min, in a gradient mode with buffer A: 0.1% aq. TFA and buffer B: 0.1% TFA in acetonitrile, monitoring at a wavelength of 214 nm which is characteristic for the amide bond.

All HPLC purifications were performed using a Phenomenex C18 preparative column (22 x 250 mm), in a gradient mode with eluent A: 0.1% aq. TFA and eluent B: 0.1% TFA in acetonitrile.

Matrix-assisted laser desorption ionization time-of-flight mass spectrometry (MALDI-TOF MS) was carried out on a Bruker Ultraflex II instrument (Bruker Daltonics, Bremen, Germany) and used to characterize the peptides at each intermediate step using sinapinic acid (3,5-dimethoxy-4-hydroxycinnamic acid) as matrix. The matrix was made up in 75% acetonitrile containing 0.1% TFA.

Analytical RP-HPLC and MALDI-TOF-MS of all compounds:

1.1 *Analogue 2 (A2)*

RP-HPLC carried out as described above using the elution gradient: buffer B 20-50% in 30 minutes, t= 18.027

MALDI-TOF-MS $[M+H]^+$ calculated 4537, 13, observed 4546.541.

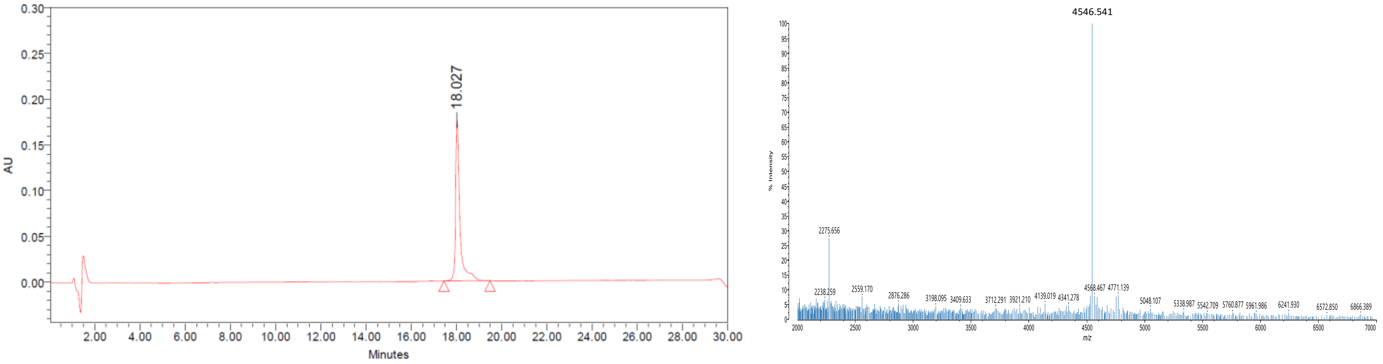

Figure S1 RP-HPLC profile of **A2** (left) and MALDI-TOF-MS (right).

1.2 *INSL5:A(8-21)T15K (Analogue 13)*

RP-HPLC carried out as described above using the elution gradient: buffer B 15-45% over 30 minutes, t=27.11min.MALDI-TOF-MS [M+H]+ calculated 4257, observed 4263.779

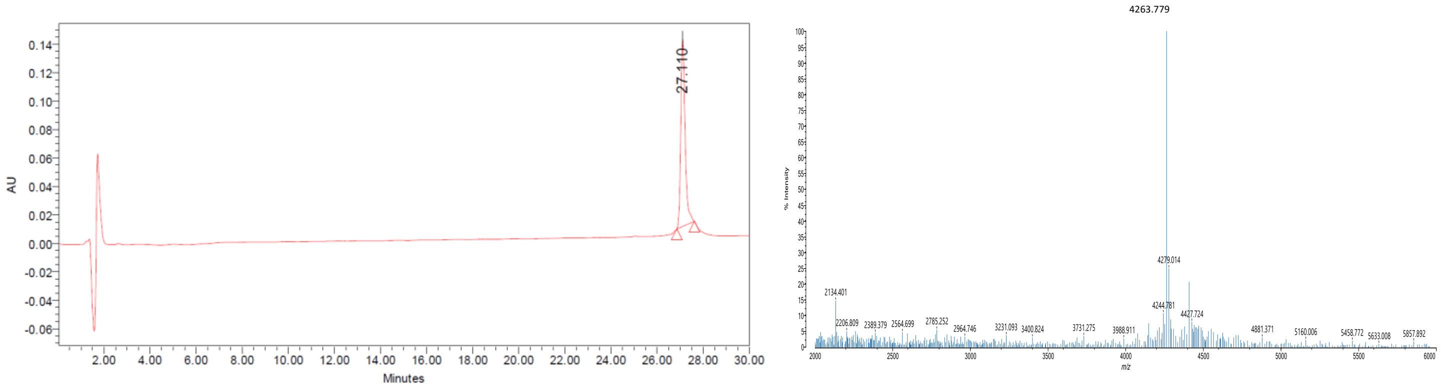

Figure S2 RP-HPLC profile of **A13** (left) and MALDI-TOF-MS (right).

1.3 ΔR3/I5

RP-HPLC carried out as described above using the elution gradient: buffer B 20-50% over 30 minutes, t =17.364 min.

MALDI-TOF-MS [M+H]+ calculated 4852.698, observed 4847.747.

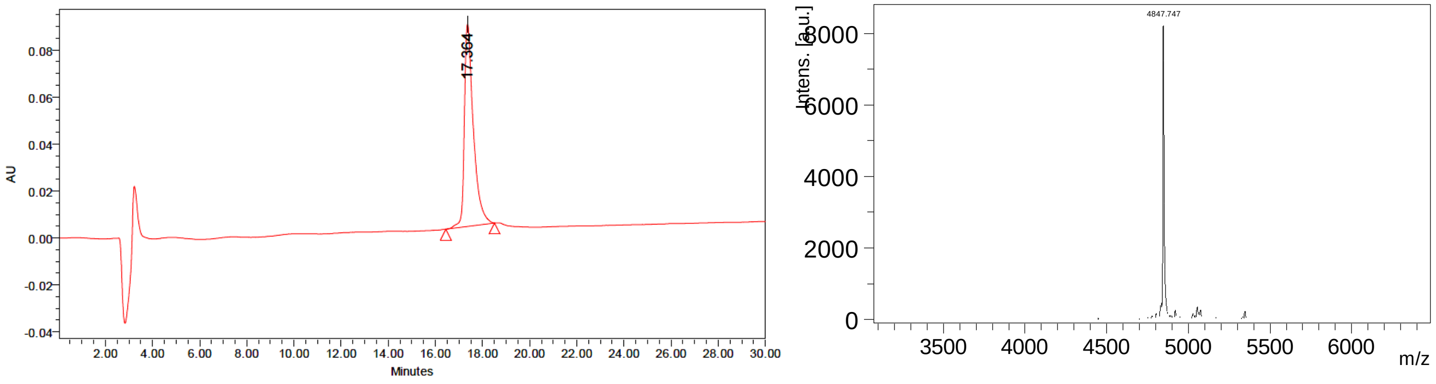

Figure S3 RP-HPLC profile of **ΔR3/I5** (left) and MALDI-TOF-MS (right).

1.4 Analogue 14

RP-HPLC carried out as described above using the elution gradient: buffer B 20-50% over 30 minutes, t=21.123min.

MALDI-TOF-MS [M+H]+ calculated 4808.69, observed 4807.084

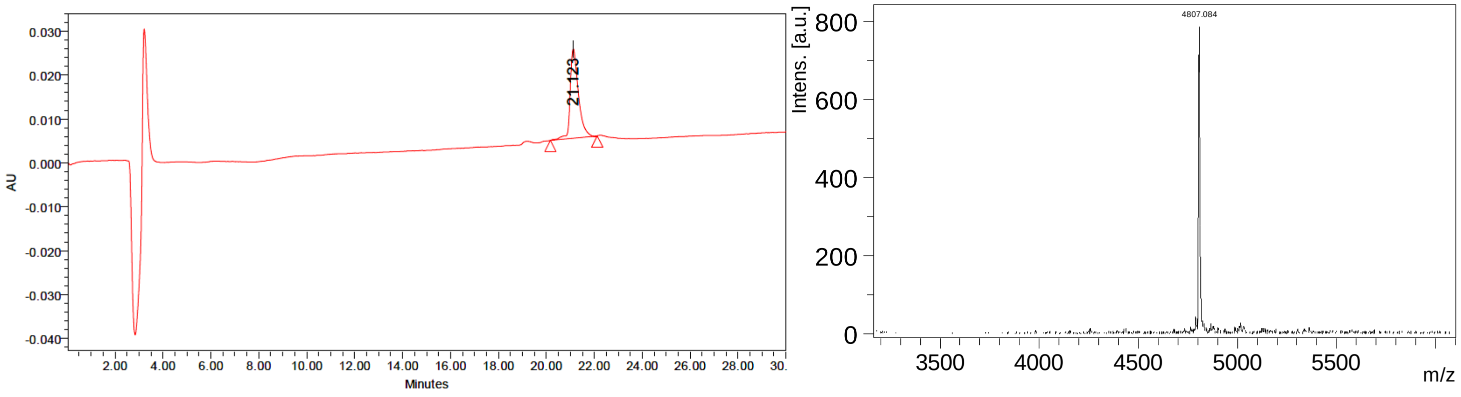

Figure S4 RP-HPLC profile of analogue **14** (left) and MALDI-TOF-MS (right).

-

1.5 Analogue 15

RP-HPLC carried out as described above using the elution gradient: buffer B 20-50% over 30 minutes, t=18.691 min.

MALDI-TOF-MS [M+H]+ calculated 4064.8, observed 4064.187.

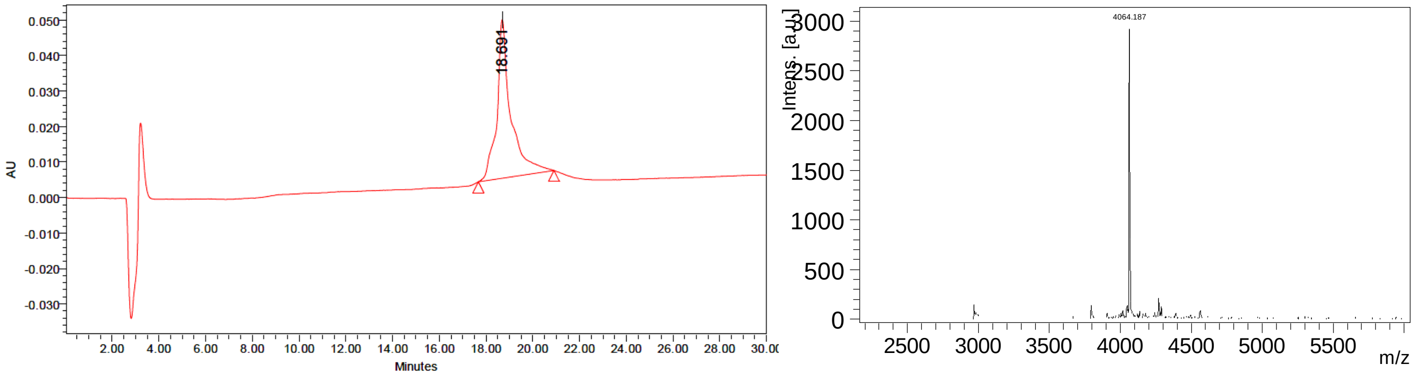

Figure S5 RP-HPLC profile of analogue **15** (left) and MALDI-TOF-MS (right).

1.6 Analogue 16

RP-HPLC carried out as described above using the elution gradient: buffer B 10-40% over 60 minutes, t=46.917min.

MALDI-TOF-MS [M+H]+ calculated 4081, observed 4084.635.

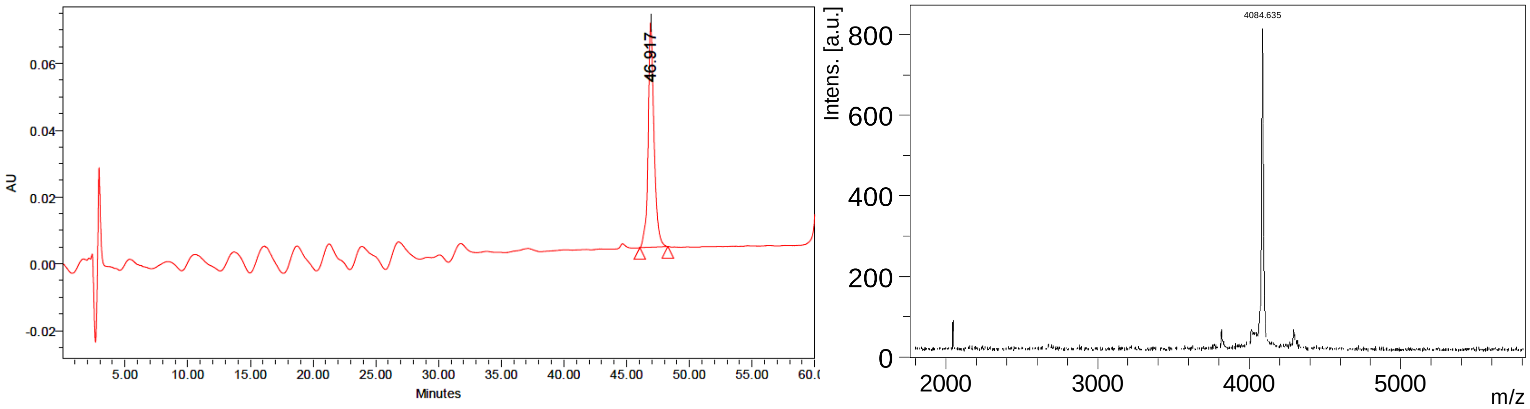

Figure S6 RP-HPLC profile of analogue **16** (left) and MALDI-TOF-MS (right).

1.7 Analogue 17

RP-HPLC carried out as described above using the elution gradient: buffer B 10-40% over 60 minutes, t=43.977min.

MALDI-TOF-MS [M+H]+ calculated 4095.813, observed 4102.015

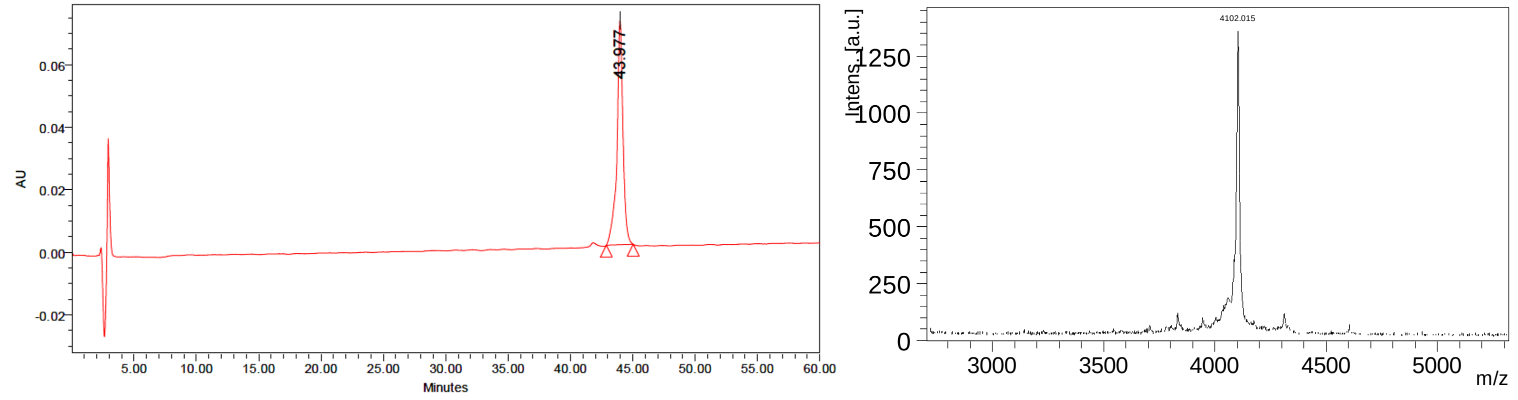

Figure S7 RP-HPLC profile of analogue **17** (left) and MALDI-TOF-MS (right).

1.8 Analogue 18

RP-HPLC carried out as described above using the elution gradient: buffer B 20-50% over 30 minutes, t=22.869 min.

MALDI-TOF-MS [M+H]+ calculated 4021.77 observed 4020.433.

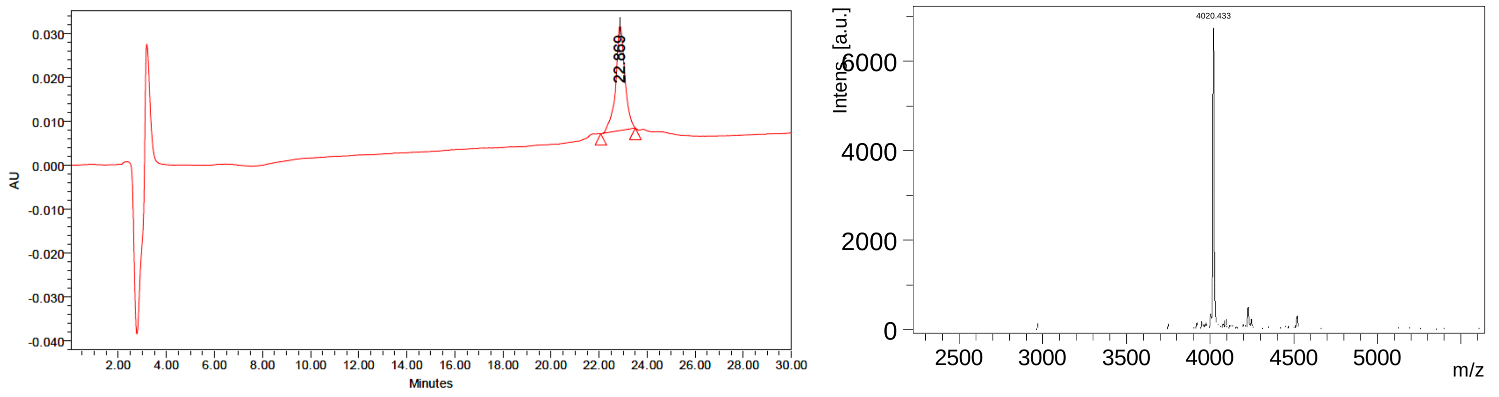

Figure S9 RP-HPLC profile of analogue **18** (left) and MALDI-TOF-MS (right).

-

1.9 Analogue 19

RP-HPLC carried out as described above using the elution gradient: buffer B 20-50% over 30 minutes, t=16.299min.

MALDI-TOF-MS [M+H]+ calculated 4096.844, observed 4102.603.

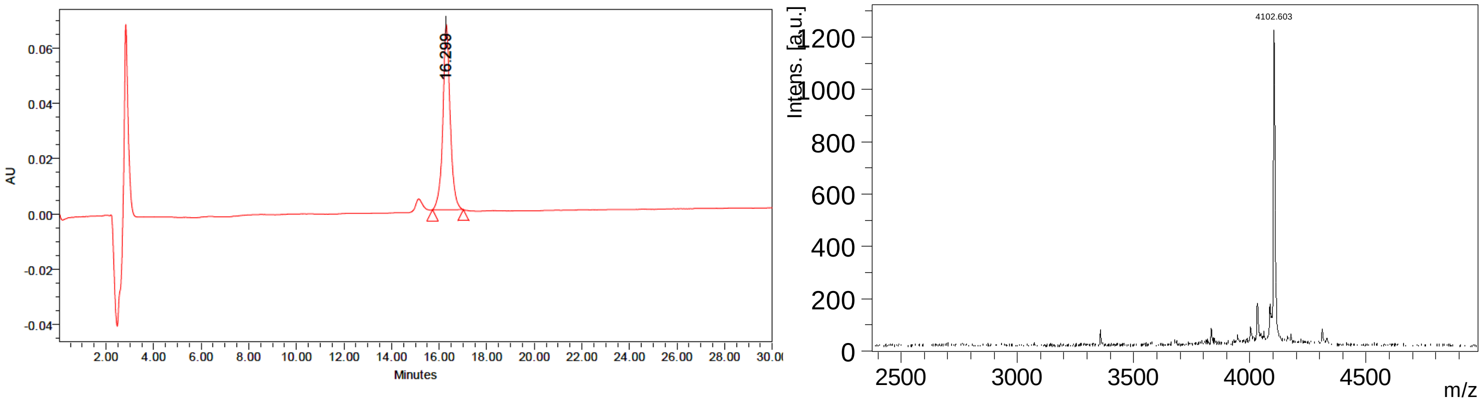

Figure S8 RP-HPLC profile of analogue **19** (left) and MALDI-TOF-MS (right).

1.10 Analogue 20

RP-HPLC carried out as described above using the elution gradient: buffer B 20-50% over 30 minutes, t=22.230min.

MALDI-TOF-MS [M+H]+ calculated 4065, observed 4066.955.

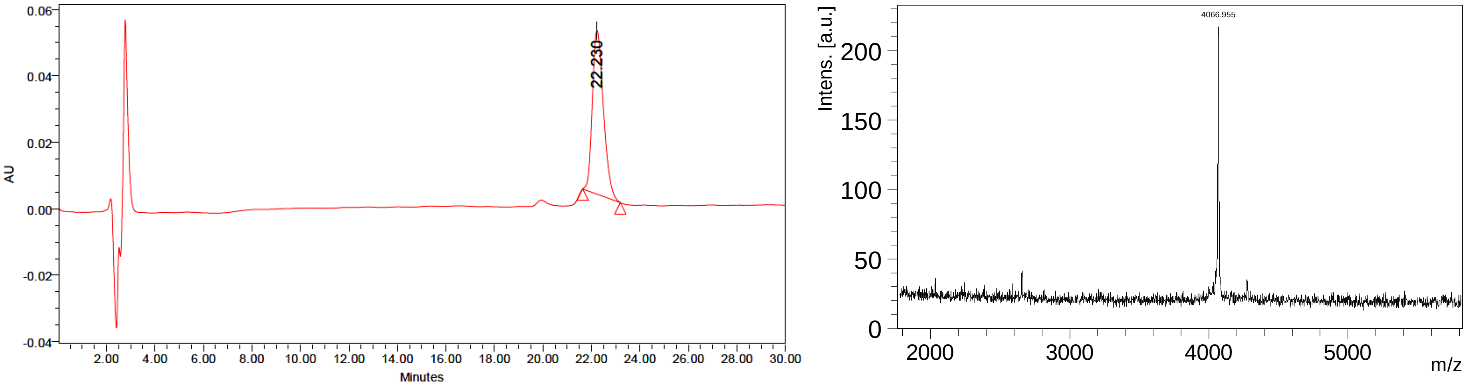

Figure S8 RP-HPLC profile of analogue **20** (left) and MALDI-TOF-MS (right).

-

2. Table S1: Summary of peptide characterization by MALDI-TOF MS and HPLC

**Analogues**	**MALDI TOF MS**		**HPLC**[2]			**Purity (%)**	**Yield (%)**
	Observed[1]	Calculat2.ed	t_R (min)	T_0 (min)	**k'** $(t_R-T_0)/T_0$		
analogue **2**	4545.262	4546.541	18.027	1.49	11.09	99.47	15.45
analogue **13**	4263.929	4263.779	27.11	1.729	14.67	99.63	14
**ΔR3/I5**	4847.747	4852.698	17.364	3.223	4.38	99.33	3.3
analogue **14**	4807.084	4808.69	21.123	3.217	5.56	93.79	20.28
analogue **15**	4064.187	4064.8	18.691	3.218	4.80	96.22	45
analogue **16**	4084.635	4081	46.917	2.932	15.00	98.88	12
analogue **17**	4102.015	4095.813	43.977	2.937	13.95	99.34	17.4
analogue **18**	4020.433	4021.77	22.869	3.186	6.17	96.49	38.1
analogue **19**	4102.603	4096.844	16.299	2.831	4.75	93.23	8
analogue **20**	4066.955	4065	46.917	2.777	15.89	97.53	10.7

[1]Observed molecular weights were determined by MALDI-TOF MS of the purified peptides

3. Peptide Yield

Analogues with three disulfide bonds:
The A and B-chains of respective peptides were separately synthesized using standard SPPS protocols. The A-chains were connected to their respective native B-chain following our protocol[1,2] to yield **ΔR3/I5** (3.3%) and analogue **14** (20.28%).

Analogues with two disulfide bonds:
The A and B-chains were separately synthesized using standard SPPS protocols. The A-chains were conjugated to the B-chain following a recently developed protocol[3,4] to give analogues **A2** (15.45%), **A13** (14%), **15** (20.28%), **16** (12%), **17** (17.4%), **18** (38.1%), **19** (8%) and **20** (10.7%).

4. REFERENCES

1 Hossain, M. A. *et al.* Synthesis, conformation, and activity of human insulin-like peptide 5 (INSL5). *Chembiochem* **9**, 1816-1822, doi:10.1002/cbic.200800113 (2008).

2 Belgi, A. *et al.* Structure and function relationship of murine insulin-like peptide 5 (INSL5): free C-terminus is essential for RXFP4 receptor binding and activation. *Biochemistry* **50**, 8352-8361, doi:10.1021/bi201093m (2011).

3 Belgi, A. *et al.* Minimum active structure of insulin-like peptide 5. *J Med Chem* **56**, 9509-9516, doi:10.1021/jm400924p (2013).

4 Patil, N. A. *et al.* Engineering of a Novel Simplified Human Insulin-Like Peptide 5 Agonist. *J Med Chem* **59**, 2118-2125, doi:10.1021/acs.jmedchem.5b01786 (2016).

-
